# Density of immunogenic antigens does not explain presence or absence of the T cell-inflamed tumor microenvironment in metastatic melanoma

**DOI:** 10.1186/2051-1426-3-S2-P425

**Published:** 2015-11-04

**Authors:** Yuanyuan Zha, Stefani Spranger, Kyle M Hernandez, Yan Li, Riyue Bao, Peter Alexieff, Jorge Andrade, Jason J Luke, Thomas F Gajewski

**Affiliations:** 1 Human Immunologic Monitoring Facility, University of Chicago, Chicago, IL, USA; 2 University of Chicago, Chicago, IL, USA; 3 Center for Research Informatics, University of Chicago, Chicago, IL, USA

## 

Immune checkpoints blockade inhibitors, such as anti-CTLA-4 and anti-PD-1, are now proven effective cancer treatments. In melanoma, the patients who experience clinical benefits usually have T cell-inflamed tumor microenvironment. Understanding the molecular mechanism explaining lack of T cell infiltration in a major subset of patients should lead to development of new therapeutic strategies that improve spontaneous T cell infiltration into tumors and render these patients responsive to immunotherapies. It has been proposed that the T cell-inflamed (hot) and non-T cell inflamed (cold) phenotypes might be due to the differential expression of immunogenic antigens. To test this hypothesis, we compared the levels of three types of antigens: cancer-testis (CT) antigens, differentiation antigens, and somatic mutational antigens, in hot and cold tumors.

Using the TCGA data set for malignant melanoma, the patients were segregated according to the presence or absence of the T cell-inflamed gene expression signature in the tumor microenvironment. Transcriptional profiling revealed no differences in the levels of cancer-testis (CT) antigens or differentiation antigens between the hot and the cold tumors. Using exome sequencing of tumor versus germline, a range of 18 to 3001 of non-synonymous mutations was observed in both cohorts. Using the syfpeithi algorithm for HLA-A*0201 patients, a median of 123 mutations having a high immunogenicity score were found in the T cell-inflamed cohort versus 176 in the non-T cell-inflamed. To confirm actual immunogenicity, 321 peptides from hot tumors and 409 peptides from cold tumors have been synthesized. Using a high-throughput T2 binding assay, peptides from both cohorts were found to bind to HLA-A*0201. In vitro priming of T cells using autologous dendritic cells also revealed that peptides from both cohorts could prime human antigen-specific CD8+ T cells. Our results indicate that there is not a lack ofimmunogenic antigens in non-T cell-inflamed melanomas. Other mechanisms must explain T cell exclusion in those cases, such as the recently described Wnt/b-catenin pathway activation. New strategies to promote T cell entry into non-inflamed tumors have a chance for success if immunogenic antigens are indeed present and can be accessed by host immune cells.

